# Perceptions of public health nursing Team Leaders (TLs) and Team Supervisors (TSs) on nurse’s development in Fiji

**DOI:** 10.1186/s12913-022-08898-z

**Published:** 2022-12-19

**Authors:** Sheenal Shivangani Singh, Masoud Mohammadnezhad, Ledua Tamani

**Affiliations:** 1 Fiji Ministry of Health and Medical Services, Suva, Fiji; 2 School of Nursing and Healthcare Leadership, University of Bradford, Bradford, UK; 3 Department of Health Education and Behavioral Sciences, Faculty of Public Health, Mahidol University, Bangkok, Thailand; 4 School of Public Health and Primary Care, Fiji National University (FNU), Suva, Fiji

**Keywords:** Nurses, Team leaders, Professional development, Fiji

## Abstract

**Background:**

Nurse team leaders are responsible for contributing to managing the quality of service delivery and facility output of their nurses to ensure there is a high quality of care delivered by the health system. This study aimed to explore the perceptions of public health nursing Team Leaders (TLs) and public health nursing Team Supervisors (TSs) on nurses’ development in Fiji.

**Methods:**

A qualitative study was used to gather information using in-depth phone interviews among TLs and TSs comprising Chief Midwifery Nursing Officer (CMNO), Director of Nursing (DON), Sub-divisional Nursing Managers (SDNMs), acting SDNMs and Nursing Manager (NM) at Central health division in Fiji. The data was collected through semi-structured open-ended questionnaires and were audio recorded. The data was analyzed using manual thematic analysis process.

**Results:**

The study comprised of 26 participants, which included 10 TSs and 16 TLs. Four themes were identified for the results amongst TSs and TLs: ethical development; professional development; psychological development; and recommendations. However, nine sub- themes were identified for TSs and eight sub-themes were identified for the result amongst TLs.

**Conclusion:**

This study highlighted that TLs and TSs elaborated on the need for the ethical, professional, psychological development, nursing development and also on the importance of policies and guidelines. Professional ethics should be integrated into the Continuous Profession Development (CPD) points that are used to renew yearly nursing licenses as well as exposing the need for having competencies on professional ethics in nurses’ logbooks. Further research is needed to determine the in-depth barriers.

## Background

According to World Economic Forum (WEF), globally there are 27.9 million nurses around the world, an increase of 4.7 million between 2013 and 2018. The WEF requested the government of Low and Lower-Middle-Income Countries (LMICs) to invest in nursing education, strengthen nurse leadership and play an influential role in forming health policies and decision-making [1]. World Health Organization (WHO) has described how increasing population leads to the health care workload whereby the manpower such as nurses is affected. Hence, more demand for nurses will also promote growth in Nurse Team Leaders in the country [2]. There are barriers that contradict nursing development such as lack of working at strategic level, limited organizational opportunities, inflexible organization, revenue, poor policy making, time demands and stress, related to nurses or leadership roles [3, 4]. Enhancing nurses’ action can’t be obtained if they are not supported by their team leaders [5].

There are several obstacles that need to be overcome before the public health approach can be in place or fully developed [6]. A study done by Pillay (2011) shows that there is a lack of knowledge and skills among team leaders to fill competency gaps for legal/ethical issues, organizing, and controlling [7]. Leadership development program provides four improvements in the health care service delivery: improving the caliber and quality of the workforce; enhancing efficiency in the organization’s education and development activities; reducing employee’s turnover; and focusing on strategic priorities [8]. Mentoring of nurses will give rise to a talented leader who can uphold leadership role at any time, and that succession planning supports developing a leader by providing sufficient leadership preparation through training, education and practical experience as part of the organizations strategic plan [9].

Nurse leaders recruited to nursing management positions need educational upgrading in order to develop a competent approach to nursing leadership. Their competency needs to be continually refined and evaluated to perform in practice and have defined competencies for nursing leadership programs [10]. British National Health Service (BNHS) mentioned that the basis for positive psychological contracts amongst nursing professionals is based on professional values and professional commitments [11]. Policies should be developed on violence prevention programs for a healthy work environment that enhances a nurse’s delivering of a quality and productive service [12].

Public health nurse works within larger communities by taking care for an entire population and work to protect its greater health. This may involve teaching the community about potential health issues, promoting good nutrition, safety, and hygiene and increasing the community’s access to care [13]. Public health nursing Team Leaders (TL) and Team Supervisors (TS) are responsible for contributing to planning, organizing, staffing, directing, controlling, regulating and reporting of their nurses to ensure that a high quality of care is delivered by the health system [14]. In New Zealand health facilities, the turbulent health care environment is impacting by day-to-day managerial issue [15]. It was also highlighted that working for long hours; workplace hazards; bullying/ harassment; shortages of staff; and dealing with advanced technologies are factors that affect nursing quality [16, 17].

The Ministry of Health and Medical Services (MHMS) of Fiji outlined that Fiji Islands has a total of 207 health facilities; 2 specialized hospitals, 3 divisional hospitals, 19 sub divisional hospitals, 86 health centers and 97 nursing stations. The MHMS of Fiji is divided into four (4) main health divisions including; Central Health Division, Northern Health Division, Eastern Health Division and Western Health Division. These are responsible for providing public health and curative health services made accessible and available to the communities on those 4 divisions [14].

The MHMS structure outlined that the division of nursing is responsible for the planning, development, coordination, monitoring and evaluation of nursing standards, policies, and guidelines and protocol to provide quality nursing care through the overarching provision of nursing technical support mechanism for quality curative and preventative health care in Fiji. In addition, the division provides management and administrative support to the Fiji Nursing Council (FNC) for the professional registration of nurses in compliance with legislative provisions in the Nursing Decree 2011 on professional registration. In the MHMS structure and workforce, the nursing profession makes up 62% of the total healthcare workforce and is virtually the face of the Ministry of Health and Medical Services to the general public. Nurses are the frontline health practitioners present in every healthcare facility level in those 207 health facilities in the country [14].

The CHD has a total of 52 health facilities; which includes two (2) specialized hospital, one (1) divisional hospital, five (5) sub divisional hospital, 23 health centre and 21 nursing stations, responsible to provide accessible healthcare services to population of approximately 383,814 in those five Medical Subdivision [14]. The structure of the nursing division in the MHMS organizational structure divides into hospital or clinical nursing and public health or community nursing. This study will focus only on TLs and TSs who are based in all the health centers in CHD. In other words, those who are based in those twenty-three (23) health centers in the CHD.

In Fiji, there are barriers and challenges with nursing development that TLs and TSs currently encounter when managing nurses. This study was conducted in Central division with the intention to determine factors (barriers and challenges) faced by public health team leaders and their working relationship with their nurses in comparison to challenges faced by nurse. Nurses need support from their team leaders to carry out their professional activities efficiently and effectively but most of the time these nurses are left disappointed without support and guidance as expected. This creates barriers and grievances between the team leaders and the registered nurses which hinders them in their working environment, hence, affecting teamwork, and a team morale and service delivery in some cases. Most of the time, these team leaders are unable to provide timely support for their staff due to factors that are not well-addressed through nursing guidelines or policies. On the same note, some team leaders are unaware of these existing work conflicts from the nurse’s viewpoint on what they have been facing and how this gap can be reduced. These challenges can be seen due to the lack of professional, psychological and ethical development amongst nurses.

To date, there is no existing literature nor was any study done in Fiji on nursing team leaders on nursing development that comes under their authority and leadership. Public Health Nursing Team Leaders have multiple priorities and with enormous pressures which really affects their day-to-day operational activities. There are factors that affect operational efficiency, decision making, ambiguity and missing data which interferes with decision-making processes. The health facility business plan contributes to the MHMS Annual Operational Plan and Strategic Plan; and works are planned accordingly to achieve the targets in these plans, however at times planning process are disrupted due to an outbreak or public health emergency alert. TLs are than required to re-adjust their plans based on the nature of the situations, higher-level turbulent issues, and resource needs.

This research study is done with the intention to determine factors (barriers and challenges) faced by public health team leaders and their working relationship with their nurses in the CHD. Understanding these factors will enable TLs and TSs to come up with more effective, efficient and practical approach to address issues facing their leadership and management issues with nurses in the division. This study will also provide platforms for future researchers and guide them to conduct studies in other divisions, for better development of nursing policies, guidelines, standard of procedures (SOPs); and updating of the nursing ACT that will be more ideal towards tackling these barriers or factors. It will also help with the transitional phase of the student nurses to nurses; the knowledge gained from nursing school and the involvement of TLs and TSs with nursing school curriculum. This information’s will help in improving the better-quality service to the nurses, team leaders, patients /community and nursing department as well. Thus, after implementing these documents by nursing unit in MHMS, nurses will get opportunities for psychological, professional and ethical development. These developments will assist TLs to provide a quality services to nurses and patients/community, hence improving the output of the business plan indicators in the health facilities, contributing to the MHMS Annual Operational Plan and Strategic Plan by successfully achieving the indicator targets as planned.

This study aimed to explore the perceptions of TLs and TSs on the nursing development and the impact to health service delivery related to the psychological, professional and ethical developments in Fiji.

## Methods

### Study design and setting

A qualitative approach was applied to explore the perceptions and experience of TLs and TSs on the nursing development in the central health division in Fiji from 26th of August, 2021 to 13th of September, 2021. There are 23 Public Health Centres (PHCs) including a school team and the women’s wellness clinic that has public health nursing team leaders in the Central Health Division (CHD) that were selected for this study. CHD has 5 medical subdivisions including Tailevu (with 3 PHCs), Naitasiri (with 4 PHCs), Rewa (with 3 PHCs), Serua/Namosi (with 4 PHCs) and Suva (with 9 PHCs). These subdivisional services are decentralized through the remote, rural and urban regions. Some of the health centres in CHD are also located in rural and remote areas however the main Central divisional office is located in an urban setting.

### Study sample

Participants in this study consisted of 26 participants who were chosen purposively including 16 TLs, and 10 TSs. Twenty-three TLs were proposed for interviews, however, 6 positions were vacant and 1 participant was unreachable, therefore 16 TLs were interviewed. There were total of 11 TSs in Central Health Division however one post got vacant recently therefore 10 PHNTL were interviewed. As for the participants for this study, the inclusion criteria included: TLs from CHD facilities and TSs including Chief Nursing Midwifery Officer (CNMOs), Director of Nursing (DON), Subdivisional Nursing Managers (SDNMs), Acting SDNM and Nursing Managers (NMs). School team and women’s wellness nursing team leaders. Those TLs and TSs who worked at subdivisional hospitals and those who were not willing to participate in this study were excluded.

Sixteen TLs and 10 TSs were interviewed until theoretical data saturation was achieved. Data saturation refers to the point in the research process when no new information is discovered in data analysis, and this redundancy signals to researchers that data collection may cease. It has further explained that saturation means that a researcher can be reasonably assured that further data collection would yield similar results and serve to confirm emerging themes and conclusions [18].

### Data collection tool

Data was collected through semi-structured open-ended questionnaires for in-depth phone interviews due to COVID-19 restrictions. The interview questionnaires were developed using the literature [19, 20], hence the questions were modified for this study by aligning them with the research questions. The questionnaires includes 24 questions for each group in 4 sections including the demographic/introduction Sect. (9 questions), barriers/ challenges Sect. (3 questions), and development Sect. (6 questions) and recommendations for the way forward (6 questions) (Tables 1 and 2). The questionnaire was developed using the English language. All data collections were carried out in the English language as all participants were well educated and confident to respond to the research questions in English.

**Table 1:** Data collection Tool for TS

Interview Questionnaire (TS)	
Barriers/Challenges	What do you think are the barriers and challenges faced by PHTL while providing assistance to Nurses in the Central Division in Fiji?
	How these barriers has effect on PHTL on the quality of services they provide to Nurses in the Central Division in Fiji?
**Developments**	What do you think about the Skills and Knowledge in Leadership Styles currently practiced by PHTL in Central Division?
	What do you think about the attitude of the PHTL towards their Nurses?
	What do you think are the challenges faced by Public Health Nursing Team Leaders on the importance of ethical, professional and psychological development of Nurses?
	What support can PHTL provide to improve the ethical, professional and psychological development amongst Nurses?
**Recommendation**	What are the recommendations on how to improve the ethical professional and psychological development amongst Nurses?
	Any Other Factors and recommendations?

**Table 2:** Data collection Tool for TL

Interview Questionnaire (TS)	
**Barriers/Challenges**	What do you think are the main barriers and challenges faced while providing assistance to Nurses in the Central Division in Fiji?
	How these barriers can be addressed?
**Developments**	What do you think about the Skills and Knowledge in Leadership Styles currently practiced by PHNTL in Central Division?
	What do you think about ethical, professional and psychological development of Nurses? Is it important or not?
	What can be done to improve ethical development amongst Nurses?
**Recommendations**	How these recommendations will help with the service delivery of the nurses?
	Any Other Factors?

### Study procedure

After obtaining ethics approval, the channel of communication was followed for recruitment of identifying potential participants through conversations with CNMOs and the Director of Nursing in the CHD, participants’ information was collected for this study. All the potential participants were asked to participate in this study.

Participants were approached by the trained principal researcher after providing a verbal explanation. Participant’s information sheet were distributed to TLs and TSs. For those who qualified in the study criteria were given one (1) week timeframe to read and understand the participants’ information sheet, participants who were willing to participate were asked to sign a consent form which were collected as evidence. TLs and TSs were assured that their information will be confidential and they were allowed to leave the study at any time.

Participants were approached through phone and were given a verbal explanation of the study. An arrangement was made about the date, time and venue of the interview. Participants were informed that the interview would be carried out in English language. The interview timeframe was arranged therefore Focus Group Discussion (FGD) lasted for an hour whereas in-depth interview for 30–40 min. Phone interview was conducted for unforeseen reasons. Due to COVID restriction protocol, all interview was through phone and due to COVID commitment for TLs and TSs, only in-depth interview was carried. All interviews were audio taped for transcription later. All the data collected and consent form received were stored in a safe locker. The locker can only be accessible to the principal researcher, and confidentiality were maintained at all times.

### Data management and analysis

All interview audio recordings were transcribed by the principal researcher and was assisted by the co-authors to make sure they were transcribed accurately. Following this, a review of transcriptions was carried out to correct errors and remove references to names, places and significant events to ensure anonymity for the participants. Once the transcriptions were clarified, data analysis was carried out. Manual thematic analysis was used to analysis the data by using six steps adopted from Braun and Clarke [21] and supported by Caulfield [22]: familiarization with the data; coding the data; generating initial themes; reviewing themes; defining and naming themes and writing the report. The principal researcher read and reread each transcript line by line, identifying similar phrases and words then assigned numbers to each word or concept. The coded data that had similar meaning was grouped together. Once grouping of similar data was completed, descriptive themes were identified to reflect the lived experiences described by the participants. 

### Study rigor

Rigor of the study is to ensure the quality is thoroughly examined for accuracy and exactness [23]. Rigorous research is research that applies the appropriate research tools to meet the stated objective. Lincoln and Guba in 1985 were the first to report rigor in their model of trustworthiness of qualitative research [24]. They stated that trustworthiness is used as the central concept in their framework to appraise the rigor of a qualitative study. The author further stated that from Lincoln and Guba’s crucial work in the 1980s, reliability and validity were replaced with the concept of “trustworthiness”. Without rigor, research is worthless, becomes fiction, and loses its use. Rigor is crucial due to its relation with the strength of the research design and the appropriateness of the method to answer the questions such as in terms of planning, data collection, analysis, and reporting [24].

In this study, to increase the rigor and trustworthiness of the study, the precision of this study was developed from early stages through a systematic search of the existing literature about the topic, from planning the layout of this study to formulating the questionnaires, to form the data collection method and analysis of data collected. The accuracy of this study was decided by continuous evaluation of thesis write up by respective supervisors. The precision of the questionnaire used was checked by the supervisor for its validity. Lincoln and Guba’s evaluative criteria were adopted to help in evaluating the rigor of this study [25].

Credibility was enhanced in terms of various engagement with each participant during the data collection process including consent forms that were obtained from the nurses to conduct the study, questionnaires used during the interview were filled in front of the participants. The semi-structured interview procedure also allowed some flexibility during interviews such as opening out of answers and the chance for requesting more information if required.

Transferability was enhanced by using the purposive sampling method to include the most appropriate participants, their life experiences for answering the research questions. Special care was given to the data collection, identification, and data analysis such as ensuring that the interview venue is quiet and the allocated time is convenient to all the participants, the questionnaires were checked again by PR prior to all online interview sessions conducted and new approaches applied when necessary to ensure relevant information acquired based on the research questions. The audiotaped data were meticulously transcribed by the principal researcher. During the analysis phase, every attempt was made to document all aspects of the analysis. After the PR categorized and was able to make sense of the transcribed data, all efforts were exhausted to illuminate themes and descriptors as they emerge.

Conformability was met by maintaining a journal during the research process to keep notes and documents anything that would be beneficial during the study. The PR maintained self-awareness of her role as the sole instrument of this study.

### Ethical considerations

Ethical approval for this study was obtained from Fiji National University (FNU), the College Health Research Ethics Committee (CHREC) with the ID- 044.21 and Fiji Human Health Research Ethics Review Committee (FHHRERC). Permission was obtained from the chief nursing midwifery officer and director of nursing central division prior to the commencement of the interview. Each participant was asked to sign a written informed consent form prior to the interview. All methods were carried out in accordance with relevant guidelines and regulations.

## Results

### Participants’ characteristics

Table 3 describes the general characteristics of 10 TSs and 16 TLs who undertook the in-depth phone interviews during the data collection period. The majority of the TSs were in the age group 41 to 50 years (50%), female (80%) and with postgraduate degrees (60%). Around two thirds of TSs (6%) worked as Sub -Divisional Nursing Managers (SDNMs) with less than 3 years’ work experience (70%). However, the majority of TLs were in the age group 41 to 50 years (69%), female (88%) and with postgraduate degrees (44%). Around 88% were confirmed PHTLs with less than 3 years’ work experience (64%).

**Table 3:** General characteristics of TSs (*n* = 10) and TLs (*n* = 16)

Characteristics	TSs	TLs
	Frequency (%)	Frequency (%)
**Age (years)**		
30–40	2 (20)	4 (25)
41–50	5 (50)	11 (69)
51 and over	3 (30)	1(6)
**Gender**		
Male	2 (20)	2 (12)
Female	8 (80)	14 (88)
**Qualification**		
Diploma	0	2 (12)
Bachelors	2 (20)	7 (44)
Postgraduate degree	6 (60)	7 (44)
Masters	2 (20)	0
**Current position**		
CNMO	1 (*10)*	*0*
Director of Nursing	1 (*10)*	*0*
SDNM	6 (*60)*	*0*
Acting SDNM	1 (*10)*	*0*
NMs	1 (*10)*	*0*
TL	0	14 (88)
Acting TL	0	2 (12)
**Work experience (year)**		
0–3	7 (*70)*	*10 (64)*
4–6	1 (*10)*	*2 (12)*
7–9	0	*2 (12)*
10–12	1 (*10)*	*1 (6)*
13–15	1 (*10)*	*0*
16–19	0	1 (6)

### Themes and sub-themes for TSs and TLs

Four main themes were present for TSs and TLs from analysing data including: *ethical developments, professional development, psychological development and Recommendation* (Table 4). In this section, TSs participants are represented with a “TS” and a cardinal number as TS1, TS2, etc. and TLs participants are represented with a “TL” and cardinal numbers as TL1, TL2 and etc.

**Table 4:** Themes and sub-themes identified for TSs and TLs

Theme	TSs		TLs	
	**Sub-themes**	**Codes identified**	**Sub-themes**	**Codes identified**
Ethical Developments	Challenges faced with ethical developments	Lack of knowledge on nursing scope of practice, code of conduct, and our nursing decree, lack of policy, Remodeling removed some process	Importance of Ethical development	Knowledge on professional ethics, Knowledge on ethical development, Enhance capacity building,
	Support to improve ethical development	Train nurses on scope of practice, code of conduct, and our nursing decree; Mentor them,, in service training on ethical development,	Recommendations improve ethical development	Promote more collaboration and teamwork, More training and awareness, Include ethic in CPD.
Professional development	Professional development challenges	Time constraint for PHNTL, Training is not presented to other staffs, Poor Budget allocations.	Importance of Professional development.	Upgrades skills and knowledge, Nurses will be future leaders, To upgrade nursing standards, Lack of knowledge enhance work conflict
	Support to improve Professional development	Identify and address what is lacking, Coaching as monitoring, evaluation of data to help in planning, In-service training,	Support to improve Professional development	more refresher training, evidence based practices are approached, Pathway Presentation after workshops
Psychological development	Challenges faced with psychological development	A lot expected from public health nurses and leaders, Counseling not done, no support psychological support	Importance of psychological development	It supports nurse mental health through counselling, Stress decreases nurses output, Provide holistic care
	Support to improve psychological development	Counselling session for PHNTL, MH Gap training is introduced business plan, Counsellors, Networking with counselling unit	Support to improve psychological development	Interact with the staffs, Approve leaves and breaks, Team leaders should give their time to nurses.
Recommendations	Enhancing workplace policies and decision making	Create SOPs at workplaces, Involve nurses in policy making, Get well verse with all guidelines or policies	Nursing standards	rotation of nurses, Nurses attached in other peripheries, Knowledge and competent with 15 nursing standards competencies.
	Management function	Involvement in planning, Setting realistic goals by planning, Identification of problems, Assessing needs, Professional ethical development needed to start in school,	Scope of nursing	scope of nursing and refresher training, professional development and training on nursing documentation
	The importance of supporting TLs and nurses	Need to feel empower in the role, Teach nurses on monitoring and evaluation and how to interpret data, enhance critical thinking		

### Themes 1: ethical developments

#### TSs perceptions

##### Challenges faced with ethical development

Participants explains that nurses and TSs have a lack of knowledge on nursing documents:

“We mostly go through the procedures, but not knowing that there are guidelines, not knowing that we do have a nursing scope of practice and I’m not sure how many of us nurses here in Fiji have a copy of nursing scope of practice.” (TS7)

Participants highlighted that challenges faced with ethical development were due to a lack of knowledge and lack of policies:

“One can be lack of knowledge, lack of policy and the policies we have team leaders don’t understand it so in order for us to implement these policies’ we need to understand and this area is a barrier in terms of ethical development.” (TS5)

However, participants also expressed how remodelling has dominated some of the essential processes such as coaching:

“One was implemented by JICA, that’s the coaching but with the implementation of MYAPA we have gone away with it slowly. We are more on business plan but monitoring of nurses is very important.” (TS9)

#### Support to improve ethical development

Participants highlighted how supports could be provided to improve ethical development through training nurses on the scope of practice, code of conduct nursing decree and by mentoring them.

*“Team leaders to do more regular training on this disciplinary guideline and they need to address nurses code of ethics, code of conduct, disciplinary action, the PSC code of conduct and values. Unethical use of social media. One thing we need to remind nurses that nursing is a profession and we have a code of conduct and we need to abide with that.” (TS4)*

Participants also highlighted that nurses should be coached, mentored and acknowledged:

*“Provide coaching, mentoring and follow up feedback to the nurses to allow the nurses to share their challenges and empowering them. Providing timely support and ensuring the availability of resources for the nurses to use. Acknowledging the nurses for their good work in an email or verbal to show the nurses and letting them know that their supervisors support them all the way.” (TS8)*

Participants suggested that a multi-sectoral approach to be taken to highlight ethical issues.

*“Arrange relevant training with professional ethics and sometimes arrange with human resource department for them to talk to our team on ethical issues.” (TS10)*

### TLs perceptions

#### Importance of ethical development

Ethical development motivates nurses to act professionally. Participants explained that ethical development should start from nursing school and not merely learnt from team leaders:

*“Should start ethical development from nursing school and not learnt from team leaders. It is shown in the service delivered, they have to be fair, welcoming and guard themselves. So that when the client leaves the client is happy with the service.” (TL16)*

Another participant mentioned that there should be regular CMEs reminding nurses about ethical development including dress and conduct codes.

*“We should have regular CMEs or short sessions reminding nurses on the ethical development especially, in terms of our conduct in the workplace, even proper uniform and how we present after hours to our society as a civil servant. So it’s very important for ethical development.” (TL9)*

#### Recommendation to improve ethical development

Participants suggested that more training and awareness should be conducted:

*“To do more training and awareness by inviting qualified personnel to train, Chief Nursing Medical Officer who is higher authority can conduct training and help us with our ethical development and also to add in school curriculum.” (TL6)*

Participants recommended that ethical development should be part of CPD in licence renewing:

*“Ahhhh… Should be part of the CPD point for licensing should have it from nursing ethical training in the CPD book... Nursing council should revise the license renewal to empower nurses on ethical development.” (TL16)*

### Themes 2: Professional development

#### TSs perceptions

##### Professional development challenges

There were many challenges addressed by TSs including those faced by the TL and nurses. Respondents mentioned that time constraint is another challenge which nurses face when it comes to professional development:

“They don’t have time. Most of the team leaders are part of the team to do the work. They are supervisors, they are supposed to supervise, upgrade themselves and teach, supposed to mentor and coach. But they can’t because of shortage of nurses.” (TS1)

Participants stated that there was bias demonstrated in the selection of nurses for training and workshops, but this has been rectified with open merit training:

“Team leaders before use to nominate who goes for training and that was positive side but sometimes they were biased and they were just picking their own staff to go for study but now it’s open merit and anyone can go which is an advantage.” (TS4)

#### Support to improve professional development

Many TSs mentioned there should be supported to improve professional development in the health system. They explained that TL should be supported to identify issues and find solutions:

*“Okay, I think the shift work nurses can’t find time to attend any proper training. So, it’s the job of a team leader, to make a program to choose nurses to come up with topics and rotate, team leaders should identify the nurses and also make a training program with them like in house, continuing program throughout the financial year. It will enhance capacity building of all the staff because medicine is something that is keep changing.” (TS3)*

They expressed the support should be provided by coaching as monitoring, evaluation of data to help in planning, and also to allow small developments in the health setting.

*“Coaching as monitoring, evaluating of data, when we present the data we say it’s a report but they don’t want to look at the report to interpret the data. If nurses know how to interpret data we will be having a good public health nurses, they will be able meet plans and forecast.” (TS9)*

Participants also explained that they should be trained on information technology so that this would help them to train their nurses:

*“Then team leaders should go through the skills of using IT thing, using of computer, using of power point presentation and all those skills. If team leaders are educated on this, they could easily support their nurses.” (TS6)*

#### TLs perceptions

##### Importance of professional development

Professional development is essential for health personnel because medical knowledge and skills require regular upgrading. Participants highlighted the need for training as part of capacity building for future leaders:

“Nurses will become future leaders of tomorrow so we need to train them to be the better leaders of tomorrow, therefore they need professional development.” (TL7)

Participants mentioned how having professional development can gain more skills and encourages further education.

“It brings the latest technique, the latest upgrade and we have to practice as a professional person, for example, providing quality care to your patients. Professional development you gain more skills and continue with the good education.” (TL8)

Participants mentioned that due to lack of knowledge, most of the time nurses are facing continuous conflict in the workplace:

“Because of the lack of knowledge, most of the time nurses starts to have conflict in the workplace so it’s important to upgrade knowledge so that they are confident on the job they are doing.” (TL3)

#### Support to improve professional development

Participants highlighted that during staff turnover’ replaced nurses should have enough training and qualifications necessary for the role.:

*“During staff turnover, trained nurse gets posted to another station so the replaced nurse should have all the trainings and qualifications e.g. EPIs, family planning before coming to one public health setting.” (TL13)*

Participants expressed the needs for extra training or workshop for the nurses and possibility of shared learning.

*“We need to do our own facility training and after the training or workshop outside the facility, participant needs to train the others to be on the same standard level.” (TL5)*

### Themes 3: psychological development

#### TSs perceptions

##### Challenges faced with psychological development

Some of the TL challenges that have been addressed by TSs by the TL and nurses included successfully achieving most of the activities set out by MHMS.:

“Public health nurse usually were overwhelmed with programs and projects, which can be very daunting, both physically and psychologically, emotionally, they’ve got so much and they cannot achieve everything, and I guess it’s a challenge that everybody has.” (TS2)

Moreover, a participant explained that there is no support provided by to the TLs:

“Nobody is there to assist team leaders, they need to have counsellors for team leaders, and these team leaders takes pressure and no one to assist them, which really contributes to their anxiety.” (TS1)

##### Support to improve psychological development

Participants stated that nurses should be encouraged to undergo psychological training and development to promote a positive mental attitude:

“Mental Health Gap training or postgraduate certificate in mental health and other small trainings was conducted on psychological or mental health training, we have to encourage our nurses to go through that to have positive mental attitude towards work, towards living, back home, and whatever activities that they participate in. (TS7)

Participants suggested for each subdivision to have their own counsellors:

“We can establish counsellors in each sub divisions or we can have one counsellor for the division to look after the psychological part of our work, like most of the nurses have social, financial problems and we need to have counsellors to provide support for them.” (TS1)

### TLs perceptions

#### Importance of psychological development

Psychological development is needed for nurses especially those working in the health sector, which demands a heavy workload and causes much stress. Participants mentioned that psychological development helps in making sound decisions:

*“Psychological development develops yourself to make proper decisions. Like, for example, if your nurses have weaknesses then support them and maturity will come within those developments.” (TL4)*

Participants also mentioned that stress decreases a nurse’s output so the psychological development is needed for them to provide holistic care to patients:

*“You have to be mentally fit in order to actually provide the best services, but sometimes when you are in stressed mode, your output will not be good and will affects service delivery.” (TL9)*

#### Support to improve psychological development

TLs recommended that counselling to be conducted with the support of other NGOs.

*“Ahhh… some counselling to be done, we have some NGO providing with the psychological counselling like in a health setting. If we have some counsellors who can come in to talk to nurses and to highlight issues that nurses needed to be counselled on.” (TL2)*

Participants explained the need to have other stakeholders to train them on the impact of psychological on their roles:

*“We need to have other stakeholders to teach us or assist us with psychological impact because we have other stakeholders who can help us with psychological effect in our nursing practice.” (TL10)*

### Themes 4: recommendations

#### TSs perceptions

##### Enhancing workplace policies and decision making

Participants highlighted the need to design workplace policies for decision-making purposes. They also stressed the importance of involving nurses in policy development:

“Try and get nurses involved in the decision making, not only in decision making, but trying to get them to be well versed with whatever guidelines or policies that we currently have in the medical field.” (TS7)

Participant suggests that Scope of Practice (SOP) for workplace to be established:

“We need to do capacity building and create SOPs in the workplaces to ensure that there is a proper communication channel for everyone, especially when there is any unusual occurrence in the workplace, or immediate need arise in the workplace, we should have proper communication channel.” (TS3)

#### Management function

Participants expressed how they believe there should be a set of management roles established to improve the service provision for nurses such as aligning theory and models with current work practice:

*“If team leaders are working systematically based on a theory or a model, you will know that there is flows of work but in reality it is not, because it’s not based on literatures, not on evidence-based learning styles, or on models or theories. Recent nurse’s graduate, they already graduated with Bachelor of Nursing but again they know so much but when they join the nursing workforce, they practice again whatever is currently being practiced.” (TS2)*

Participants suggested that currently supervisors are unaware of the curriculum in the school of nursing and should be advised on this:

*“We don’t know what nurses are learning in school, supervisors should be informed of the curriculum in nursing school, and training and also proper orientation during their induction, professional ethic should be part of their logbook competencies.” (TS4)*

#### The importance of supporting TLs and nurses

Participants explained TLs need to be supported by being empowered through mentoring and also to training nurses because they are the future TLs:

*“Team Leaders need somebody to mentor them, and they need to understand their role description very well, not only the role description but also the subject that comes under that.” (TS4)*

Participants explained that nurses should be involved in interpretation of data and in monitoring and evaluation so that they can plan well:

*“Teaching nurses on monitoring and evaluation, and how to interpret data. Using of Microsoft, excel need to be understood by nurses. Ethical development will enhance critical thinking.” (TS9)*

### TLs perceptions

#### Nursing standards

Participants explained that nursing standards need to be maintained at all times and furthermore, that nurses need to be rotated as well as be attached to other units. They also suggested that nurses be encouraged to have a sound knowledge of standard competencies:

*“Nurses should be rotated and attached in other units such as O&G, foot care, family health to upgrade the knowledge and skills of the nurses, since they are all graduated with general nursing for 3 years and attachment will help them in refreshing and upgrading of their knowledge and skills.” (TL1)*

Participant explained that nurses should undergo regular staff rotation training on nursing standards and too, on the fifteen (15) competencies:

*“Nursing standard that we have really need to get through to the nurses, we can have regular rotation of nurses, develop the nurses on the nursing standard and 15 standards that public health nurses should have so all team leaders should be well equipped in order to get the nurses to understand the nursing standards. This standard has all the competencies that a public nurse should know and understand.” (TL9)*

#### Scope of nursing

Participant explained that there should be training on scope of nursing and refresher training as well as conduct induction sessions for new graduates to have to remind them that nursing is a profession:

*“Training on our scope of nursing, refresher training for nurses and all those coming in as new graduate to have induction and to remind them that nursing is a profession.” (TL6)*

Participant recommended on professional development and training on nursing documentation:

*“We should have professional development and training on the scope of practice and also for the competency and standard for registered nurse which will be measured in their output, the work they do, unit plan, or quarterly report.” (TL11)*

## Discussion

Ethical development is very important for the nursing profession but there are so many challenges faced in this area. The findings from this study highlight major challenges that were faced with ethical development: lack of policies, confidentiality and lack of knowledge on the nursing SOP. The study has also highlighted the importance of ethical development to motivate nurses to act professionally and ethically. Participants explained that there is a lack of knowledge on ethical development and professional ethics amongst nurses and TLs. There should be professional ethics which includes aspects such as appropriate dress code, punctuality, capacity building and regular CMEs. Engaging of experts, provision of ethics literature and code of ethics are helpful for ethical reasoning and decision making in gaining new dimensions and outside knowledge [26]. The new and evolving leaders should include robust opportunities to identify and resolve ethical dilemmas during their professional development. There are two sets of ethical dilemmas faced by nurse leaders as they encounter inherent tensions between patients’ rights and responsibilities; and managing sparse resources in a working environment [27]. Finding from this study highlighted that nursing procedures and guidelines were not followed and most of the nursing professional in Fiji did not keep a copy of nursing scope of practice. It was also identified that there were lack of awareness on the nursing documents such as code of conduct and other nursing documents, some nurses at times practiced unethically out of ignorance because they did not have the knowledge. Leadership at times is imprecise and filled with unknown challenges, and these challenges can be addressed when educators create learning opportunities to identify and explore the types of ethical dilemma being faced to enhance their ethical reasoning skills to deal with these complex ethical issues [27].

Ethical aspect is not well addressed due to lack of policies and knowledge on policies where by TLs do not understand these policies in order for them to implement. In ethical development, confidentiality is very crucial and important. Nurses are unaware of confidentiality e.g. uploading of patient’s photos on social media. There should be more awareness created on ethical development like confidentiality, dress code, punctuality, etc. Regular meetings or coaching should be conducted by TLs, however, the remodelling of MHMS has implemented a system to monitor the nurses 6 monthly (MYAPA and business plan), hence this has dominated the procedure for coaching which was practiced previously and effective as well. The suggestion provided was that there should be regular supervisory visit by TSs to enhance capacity building of nurses and TLs on ethical development.

The main support to improve ethical development is through the training of nurses and TL on the scope of practice, code of conduct, nursing decree, nursing guidelines, policies and other nursing documents. Other recommendations suggested to improve ethical development were teamwork; create awareness; conduct social gatherings; hold regular meetings; develop capacity; conduct training; make provision for compulsory yearly ethical development assessment; develop ethical development policies, procedures and guidelines; and introduce ethical development in nursing school to be part of the curriculum. Nurse Managers should implement ethical guidelines on leadership, Furthermore, as nurse educators should be enlightened on leadership development for knowledge and skills [5]. The importance of a professional history is undeniable in terms of the formation of a professional culture and professional consciousness as well as professional ethics. It is necessary to include ethics and history issues more comprehensively in nursing curriculum. It is thought that the history of nursing and ethics department will contribute to increase the quality of education and studies in the field [28].

Tertiary institutions and health care organizations should plan nurse manager education programs which focus on strategic issues, leadership knowledge; managing difficult situations; change management; work unit management and wellbeing [20]. Some barriers for professional development were lack of time; staff shortages; limited opportunities for training and limited budget. Successful management requires skills, competencies, support and teamwork [29]; Moreover, leadership development programs empower nurse leaders to increase their self-confidence and bring positive stylistic changes in their management [30]. An effective mentorship relationship can retain staff, further develop experienced nurses, and aid in succession planning. This relationship allows nurse to have one on one time with an experienced nurse to discuss things such as career goals, work stressors and how to overcome them, and time management. This type of relationship has a positive impact on staff resilience and can boost engagement and retention of nurses. Therefore, nurse leaders have the responsibility of providing resources and support needed by nurses to promote professional development. Some of the ways to achieve this are through motivational interviewing, bringing education to nurses, encouraging shared governance, promoting self-care, and creating a mentorship program [31].

The support for professional development included the established health system; upgrade knowledge of new management techniques; evidence- based practice: regular training: capacity building: teamwork: interpretation of data: planning and implementing new ideas: ongoing CNE: training on technologies: nurses’ attachments in clinical settings: refresher training: and evidence-based practice. Developing skills and abilities to enhance managers level of technical expertise practice, loyalty and engagement, staff development is comprehensive than that of leadership skills, suggesting the opportunities for specialized training or advance education [32]. Other recommendations were for staff turnover, with nurses who take over should being well-trained in all areas so that service delivery; all training and participants should present to the unit on completion to maintain the nursing standard and theoretical and evidence-based education. The health care organizations responsibility is to set-up a clear vision and goals and make a supportive atmosphere for the successful Nurse Leadership [20].

The study findings showed major challenges for nursing development such as workload and stress; a busy and pressurized environment; financial issues and lack of investment on staff’s health and wellbeing, no proper counselling or psychological support provided to nurses and TLs however a counsellor should be provided to each subdivision. Similar study was done to improve issues such as rewards given for innovation and empowers nurses to carry out their work effectively [33].

To support nurses a strategic approach that applies effective communication; innovation; and analysing and evaluation of programs after implementation are needed. Adopting a strategic approach will require staff involvement to help in overcoming f barriers to initiating a wellbeing program [34]. The support provided is to improve nursing development, includes regular counselling sessions for nurses and TLs; training on MH gaps aligned with a business plan; team building; proper networking with counselling units; and resources provided to TLs. For the nurses with mental health, multisector approach to be provided with counselling; for each subdivision to conduct 6 monthly, quarterly or fortnightly counselling; all nurses to undertake in-services training on stress, anxiety, depression and suicide prevention; attachment in other peripheries; and on having knowledge and skills on 15 nursing competencies components.

The nurse leaders should be encouraged to participate in, mentoring, supporting and developing future nurses through developing policies. Having knowledge and skills in health policy development should be supported through role models, supportive mentorship, networking and experience. This study found barriers for health policy development such as; lack of nurses’ involvement and support, negative image of nursing to job, lack of enabling structures and lack of available resources [35]. Factors that affect policy development were: political factors: gender issues: a lack of public understanding; financial issues and resource limitations: skills training in policy development; leadership competency; and the ability to use research to influence policy makers [36].

Some of the recommendations made included to have a succession plan, skills, knowledge and experiences on leadership: to conduct weekly meetings to discuss improvements; build capacity have a process whereby vacant positions are filled for the empowerment of nurses and TLs, and work systematically, based on a theory. The character of work performed affects leaders and nurses by causing burnout, therefore nurses at different stages of career development require different types of leadership skills [37]. Other recommendations made were that nurses should be given leave on time to avoid work stress, being overworked, exhaustion and burnout; equip TLs with proper technology; for ethical development to start from nursing school and which should be part of a competency logbook competencies; supervisors to be aware of the nursing curriculum, supervisory visits; and for nurses to be involved in data gathering and monitoring and evaluation of programs.

The study suggested that nursing standards need to be maintained by nurses at all times and nursing staff need to be rotated and should be attached in other peripheries in order to be updated and upgraded with new knowledge and skills. Normally nurses understand general nursing knowledge, however, this study recommended that they be attached to other health settings. This will enable them to be upgraded with new knowledge and skills and gain a better understanding of communities’ health needs. All nurses should undergo training on SOP so that they are mindful and fully aware of nursing roles. Again, nursing competencies have 15 components which nurses need to know and be well versed at all times.

### Study limitations

The limitation of the study was that the principal researcher was unable to conduct face-to-face interviews due to COVID-19 restrictions, and thus conducted them instead via phone. There were vacant positions and some public health TLs were involved in the vaccination drive. The study was small-scale, and caution should be exercised in generalizing from these findings.

## Conclusion

This study has identified ethical, professional and psychological development challenges faced by nurses and recommends strategies and policies to overcome them in the coming years. This study showed that there was a gender biased term lack of training and development amongst nurses and TLs. Due to inadequate leadership knowledge and skills, TLs were faced with challenges in providing the support to nurses. Other contributing factors included a shortage of staff which in turn gave rise to workload and stress experienced by the nurses, which in turn affects professional development and mental health of nurses Non-compliance with nursing standards, affected ethical development, was being identified in this study. Recommended interventions such as regular training and upgrading of knowledge and skills on management; team building; capacity building; motivating and mentoring of nurses; nursing practices to be based on theory; evidence-based practice; the introduction of professional ethics in the nursing school curriculum; workplace SOP; the introduction of policies and guidelines; involving nurses in policy making; involving nurses on proper planning, data interpretations, monitoring and evaluation process; regular counselling; and other necessary support were identified.

## Data Availability

The data that supports the findings of this study is available on request from the corresponding author.

## Abbreviations

BNHS: British National Health Service
CHD: Central Health Division
CMNO: Chief Midwifery Nursing Officer
CHREC: College Health Research Ethics Committee
CPD: Continuous Profession Development
COVID-19: Coronavirus disease 19
DON: Director of Nursing
FHHREC: Fiji Human Health Research Ethics Review Committee
TLs: Public Health Nursing Team Leaders
TSs: Public Health Nursing Team Supervisors
NM: Nursing Manager
PHCs: Public Health Centres
SDNMs: Sub-divisional Nursing Managers
WEF: World Economic Forum
WHO: World Health Organization.
